# Post-Processing of Raw Data Recorded Continuously Using a FORS—Fibre-Optic Rotational Seismograph

**DOI:** 10.3390/s22228673

**Published:** 2022-11-10

**Authors:** Bartosz Sakowicz, Marek Kamiński, Michał Dudek, Anna T. Kurzych, Leszek R. Jaroszewicz

**Affiliations:** 1 Department of Microelectronics and Computer Science, Lodz University of Technology, 221 Wólczanska St., 93-005 Lodz, Poland; 2 Institute of Applied Physics, Military University of Technology, 2 gen. Sylwestra Kaliskiego St., 00-908 Warsaw, Poland; 3 Elproma Elektronika Sp. z o. o., 2A Duńska St., 05-152 Czosnow, Poland

**Keywords:** data gathering, rotational seismology, fibre-optic rotational seismograph, data analysing, automatic data improvement, filtering

## Abstract

Modern optoelectronic devices use the advantage of digital systems for data processing aimed at delivering reliable information. However, since commonly used DACs have limited accuracy, some artefacts can be observed in data streams, especially in systems designed for continuous, long-term process monitoring. In this paper, the authors’ experience with data enhancement using a fibre-optic rotational seismograph (FORS) operating in a closed-loop mode is presented and discussed. Generally, two kinds of enhancement are described. The first one uses suitable filtering techniques adequate for FORS noise investigation, as well as a suitable data resampling method for transmitted data file size reduction. The second one relates to the artefacts observed during data recording in real time. The recording starting point is triggered when the detected signal exceeds a middle signal level and, therefore, the existence of artefacts generally disturbs the recording process. Although the artefacts are easily recognised by human eyes even at first sight, their automatic elimination is not so easy. In this paper, the authors propose a new concept of signal filtering to solve the above problem.

## 1. Introduction

Non-telecommunication fibre-optic systems are of growing interest in this century, especially for sensor application. Depending on the accuracy or sensitivity required, they can be divided into two classes of systems. The first one is potentially cheap in-line structures using the lab-on-fibre concept [1]. This concept is dedicated to novel and highly functionalized technological platforms completely integrated in a single optical fibre, which would allow the development of advanced devices, components, and sub-systems to be incorporated in modern optical systems for communication and sensing application. These platforms use any kind of optical fibre, including classical single- (SMFs) or multimode fibres (MMFs), polarization-maintaining fibres (PMFs), microstructured optical fibres (MOFs) [2,3], as well as plastic optical fibres (POFs) [4,5]. Since the light beam is ‘protected’ from external fields in each optical fibre, the sensor application needs a special method to secure the guiding light interaction with a given external field. This is done by inscribing different grating structures in the fibre [6,7,8], tapering a biconical fibre [9,10,11], cutting one side of the fibre [12,13], or manufacturing special tips on the fibre facet [14,15,16]. Next, the external measurements of physical or chemical quantities are detected via changes in the refractive index, which can be expanded by the application of suitable functional materials. The second class of systems are extremely sensitive sensors based on interferometric configuration [17]. Although fibre-optic interferometers are used to detect various physical parameters, including temperature, strain, pressure, and refractive index, they are most interesting for detecting nonreciprocal physical phenomena, such as the Kerr effect [18], the Faraday effect [19], and especially the Sagnac effect [20].

The fibre-optic gyroscope (FOG) using the Sagnac effect [20] is probably the best-known practical implementation of a fibre-optic sensor with extremely high technical parameters that are unachievable by other technologies. For example, the inertial grade system requires an angular random walk (white noise) below 0.005 deg/√h, a bias drift below 0.01 deg/h, and a scale factor accuracy in the range of 5 ppm [21]. To achieve such parameters, a special optical, as well as a signal processing technique, must be applied. After more than 40 years of development of FOG technology, the minimum optical configuration [21] and the all-digital closed-loop approach proposed late 70s of the previous century by Arditty et al. [22,23] still seem to be the best solutions in this matter. Unfortunately, the practical realizations of such devices are very expensive, and are available mainly for military applications and proposed by a limited number of companies.

On the other hand, the development of the so-called rotational seismology (RS) as a new emerging field studying all aspects of rotational ground motions induced by earthquakes, explosions, and ambient vibrations [24] opens a new direction for systems based on FOG. The main difference between a fibre-optic rotational seismometer (FORS) and a FOG is its recording of angular rotation rate instead of angle changes [25]. It seems that a FORS uses the Sagnac effect directly without the need for time integration of the output signal as it is in a FOG, and, thus, the drift problem is not a limited parameter for a FORS as it is for a FOG. Furthermore, the general RS requirements for both seismic [26] and engineering [25] applications require mobile, autonomous devices which can detect rotational motions in the frequency range of 0.01–100 Hz with an amplitude ranging from about 10^−8^ rad/s for a seismic application up to a few rad/s for an engineering application. Moreover, the field of seismograph application requires a network of devices that secures the continuous monitoring of rotations over a very long time, generally for years, with automatic data recording with precise identification of events during the time of existence, as well as precise location of devices ([27], pp. 261–308). A FORS based directly on a FOG with an all-digital closed-loop architecture appears to be the best solution to fulfil all the above requirements, as shown, for example, by a blueSeis-3A device (iXBlue, Cedex, France) [28] and our previous solution FOSREM (MUT, Warsaw, Poland) [29]. However, field exploitation, especially related to the common testing of many different rotational seismometers at the end of 2019 [30,31], shows that the raw data obtained from such devices should be properly processed before their future practical use and analysis.

In this extended paper, which is intended for a presentation at the 9th International Symposium on Sensors Science I3S 2022 (20–22 June 2022, Warsaw, Poland), the research on the proper processing of raw rotational seismic data prior to their future use, based on the authors’ experience, is presented. This experience was gathered over 15 years during the development of four classes of FORSs: starting with an autonomous fibre-optic rotational seismometer (AFORS) [32,33] through a fibre-optic system for rotational events and phenomena monitoring (FOSREM) type SS [25,29] and type BB [34,35], and up to fibre-optic seismographs (FOS5s) [30,36,37].

Generally, two kinds of enhancement are described based on a short description of the latest FOS5 device. The first one uses appropriate filtering techniques adequate for FORS noise investigation, as well as appropriate data resampling to reduce the transmitted data files. The second is related to the artefacts observed in-real time data recording. Since the signal noise above the mean level is used as an initial factor of recording, the occurrence of artefacts in general disturbs the recording process. Although the artefacts are easily recognised by human eyes even at first sight, their automatic elimination is not so easy.

## 2. FOS5—Fibre-Optic Rotational Seismograph Construction

**Figure 1:**
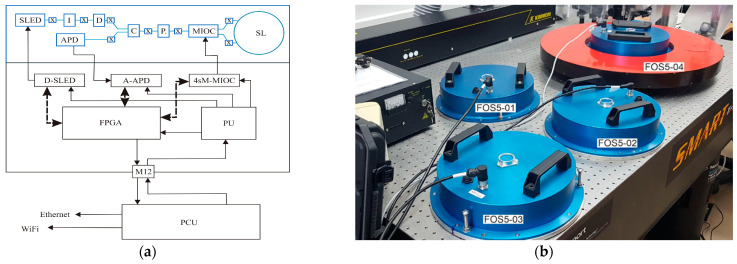
Scheme of the FOS5s (**a**): top–optical part and bottom–electronic part, and (**b**) view of the FOS5-01, …, FOS5-04 in the laboratory of the Military University of Technology, Poland [37].

The main experimental data presented in this paper were obtained from the FOS5s, which meet all the RS requirements. They are newly developed fibre-optic interferometers for a high-resolution readout of the Sagnac phase shift [20] induced between two counterpropagating waves in a closed optical path when the plane of propagation undergoes angular velocity [21,38]. The general construction of the FOS5s can be divided in two parts which operate dependently: optical and electronic. The optical part is based on the minimum gyro configuration with two types of sensor loop (SL): the FOS5-0X (X = 1, 2, 3) uses a 5000-m long SL with a 0.25-m diameter, whereas the FOS5-04 has a 14,400-m long SL with a 0.61-m diameter. It should be noted that the FOS5-04 uses the same sensor loop as the AFORS [32,33] and, in the literature, it is sometimes treated as a giant interferometric FOG [39]. All FOS5s are based on a single-mode optical fibre SMF-28e+ (Corning Inc., New York, NY, USA) and a multi-integrated optical circuit (MIOC)-(IdealPhotonics Ltd., Kwun Tong, Kong Kong), and also use other telecommunication fibre-optic components, including an isolator (I), a depolarizer (D), an X-type coupler (C), and a polarizer (P) that are coupled together via fused splices (X) in the structure shown in Figure 1a. A super-luminescent diode (SLED) was used as the light source with an optical power of *P* = 10 mW, a central wavelength of λ = 1311.2 nm, and a bandwidth of 51.2 nm (Exalos AG, Schlieren, Switzerland), whereas an InGaAs avalanche photodiode APD-1310 (Opotoway Technology Inc., Hukou Township, Taiwan) was used as the detector (APD). In contrast to our previous works [25,29,32,33,34,35], the FOS5s operate in an all-digital closed-loop configuration [22,23], which is realized by a new electronic part (see Figure 1a). The electronic part consists of the following modules: a SLED driver (D-SLED) (Exalos AG, Schlieren, Switzerland); a four-step modulator for MIOC driving (4sM-MIOC); an analogue amplifier for APD (A-APD); control modules based on FPGA (for processing the sensor data and performing necessary calculations and filtration); and a power module (PU) that uses a 24 VAD/20 W via a power and communication unit (PCU). This internal digital processing unit provides rotational speed value directly in a digital form. The FOS5s have an RS-485 interface for data and a USB interface for diagnostics (see Figure 1b). The connection provides data transmission and power supply from the PCU over only one standard M12 cable within the distance of 500 m. The PCU is connected to the Internet via an Ethernet, WiFi local networks, or 3G/4G mobile networks. The system provides the VPN functionality; thanks to this, one can connect multiple sensors in one large, synchronized network and monitor many sensors from an optional location. The FOS5s operate at a 1-ms sampling rate which secures data transfer at 1000 sps.

A different SL size has direct influence on the sensor’s sensitivity. For the FOS5-01, -02, and -03, total transmission losses are in the range of 19.20–21.60 dB, given a theoretical sensitivity of about 6.82 × 10^−8^ rad/(s√Hz), whereas the FOS5-04 with an optical loss of 17.41 dB has a theoretical sensitivity of about 1.14 × 10^−8^ rad/(s√Hz) which is approximately 6 times better.

The whole idea behind the construction of the FOS5s was to design a portable, remote, and resistant to external conditions sensor. Moreover, in order to apply the sensor in all environmental conditions, it is hermetically sealed and equipped with waterproof connectors meeting the IP67 requirements (see Figure 1b).

## 3. Results

The experimental data were collected mainly during a field exploitation of the FOS5s. The data for the FOS5-01, -02, and -03 were collected during a special experiment organized as “Rotation and Strain in Seismology: A comparative Sensor Test”, which took place in the Geophysical Observatory in Fürstenfeldbruck, Germany, between 18 November and 20 November 2019 [30,31]; during the engineering structure investigation in Warsaw, Poland, between January 2020 and August 2021 [36]; and in a historic coal mine “Ignacy” in Rybnik, Poland [37]. The main experimental data for the FOS5-04 were collected in the Seismological Observatory of the Polish Academy of Science (PAS) in the underground of Książ Castle at the Silesia region of Poland, where the copper ore deposit exploitation in this area is accompanied by a high level of seismic activity [32,37].

### 3.1. Investigation and Processing of Device Self-Noise

Recorded seismic signals always contain noise, and it is important to be aware of both the source of the noise and how to measure it. Noise can have two origins: noise generated in instrumentation and ‘real’ seismic noise from earth vibrations. Typically, the instrument noise is much lower than the seismic noise and is difficult to measure, especially in laboratories located in research institutes with a high level of urban noise. For the above reason, the first step of the FORS investigation was to measure and analyse the so-called FORS self-noise level. The FORS self-noise is the output signal of the sensor when the sensor is at rest and there is no rotational motion at the input. It determines the lower limit of the FORS resolution. Estimating the FORS self-noise requires a recording from a seismically quiet time span with a background level of ground motion lower than the sensor’s self-noise level. One excellent opportunity for analysing the output of the sensor at rest is during night-time recording at a given location (especially at a seismological observatory). Bernauer et al. [26,40] adopted a method that was originally proposed by Sleeman [41] for measuring the self-noise of traditional seismic sensors and digitizers to estimate the self-noise levels of FORSs. Although the noise is characterised by a power spectral density (PSD), the FORS self-noise is commonly reported as an amplitude spectral density (ASD) which is the square root of the PSD. The main reason of such approach is the possibility of a direct comparison of its level to the FORS-measured amplitude of rotational motion.

**Figure 2:**
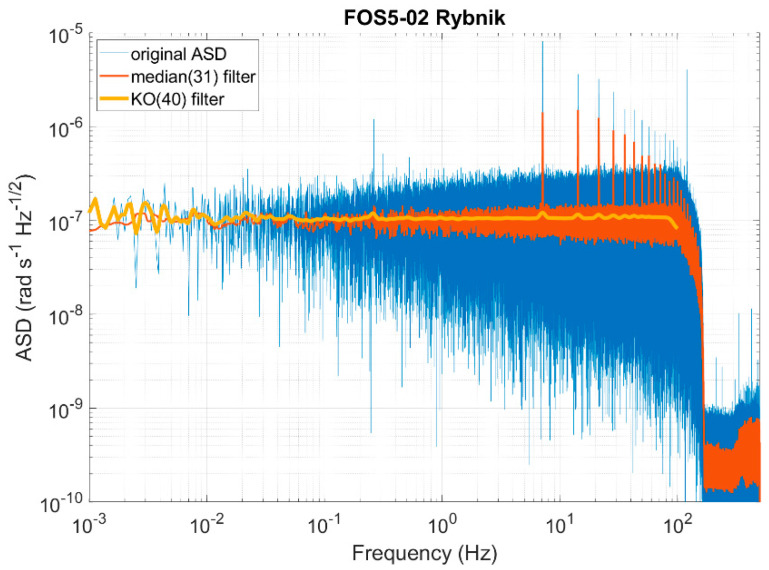
Investigation of self-noise level: the measured ASD raw data (blue line), the raw data filtered with a median filter of the order of 31 (red line), and the raw data filtered with the Konno–Ohmachi filter with a smoothing coefficient of 40 (yellow line) for the FOS5-02 [37].

Figure 2 shows an example of the ASD obtained using the FOS5-02 during the self-noise investigation at the coal mine “Ignacy” in Rybnik, Poland, registered on 28 August 2021 [37], where the raw data are presented in blue. As can be seen, the raw data are of a “fluctuating (noisy)” nature with the amplitudes increasing with higher frequencies. Such data must be post-processed to improve the results of a later analysis. The median filter, which is a non-linear digital filtering technique, is often used to remove noise from an image or signal [42] because, under certain conditions, it preserves edges while removing the noise. However, it uses the same smoothing window widths at all locations, which is not a proper approach for logarithmic data. That is why the Konno–Ohmachi smoothing algorithm [43] is preferred, which achieves a “uniform-span” smoothing of the frequency spectra on a logarithmic scale. For lower frequencies, the Konno–Ohmachi smoothing window is narrower (i.e., less smoothed), and for higher frequencies, the window is wider (i.e., more smoothed). This feature is preferred because the changes in amplitudes in lower frequencies (<10 Hz) are more important than in higher frequencies. As one can see (Figure 2), the two filters—median and Konno–Ohmachi—yield similar results only for a narrow range of frequencies; for lower frequencies (below 0.005 Hz), the median filter over-smooths the raw spectrum and, for higher frequencies (above 0.1 Hz), it does not provide enough smoothing, which is undesirable. The median filter’s response is highly dependent on the size of the smoothing window—in our case, it was set to 31 (red line). For the reason presented above, the calculated self-noise of the FOS5-02 as ASD characteristics was filtered by means of the Konno–Ohmachi filter with a smoothing coefficient equal to 40, which is generally used in seismology as the default and sufficient one (yellow line, Figure 2). The applied filtering ends at 100 Hz do not cover the entire ASD characteristics presented, because the Konno–Ohmachi filter is highly time-consuming due to the varying window widths, especially for higher frequencies. However, the frequencies above 100 Hz are not so important based on two aspects discussed below.

Such an obtained self-noise of the FOS5-02 shows almost flat characteristics of the device in the desired range of frequency (0.01–100 Hz), although with a sensitivity of about 47 percent above the theoretical value. Moreover, the two characteristic features are observed which originate from how the electronic part works.

The first is the ASD characteristic cut above 200 Hz. It is related to the electronic part design. Since the frequency range expected for the RS is up to 100 Hz, the electronic part uses analogue digital converters (ADCs) optimized to this value and low-pass filters up to 200 Hz according to the Nyquist–Shannon sampling theorem [44].

**Figure 3:**
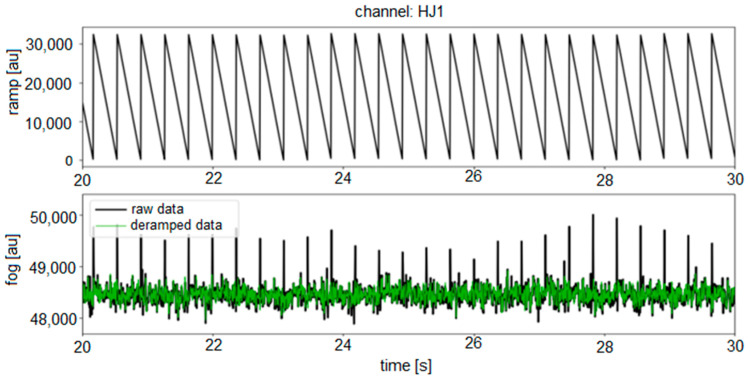
Example of a deramping procedure protected against the effective removal of ramp peaks from the raw data: the top window is an example of the ramp signal driving the MIOC, and the bottom window is a simulation of the raw and deramping data using the Bernauer script [46].

The second characteristic is the additionally registered, low-amplitude regular sharp peaks observed above a frequency of about 7 Hz, with their amplitudes decreasing with higher frequencies. As described by Murray-Berquist et al. [45], these are the characteristics of all FOGs using an all-digital closed-loop processing, and they are caused by “ramp peaks”. The electronic part uses a ramp voltage to counter act rotations. This ramp voltage increases and then falls back to zero each time it reaches 2π. The frequency at which 2π is reached depends on the rate of rotation detected by the sensor. This creates a periodic but not perfectly sinusoidal signal, which causes a large peak at the main frequency of the signal and harmonics towards higher frequencies. For a FOG, where angle changes are detected as an integrated signal of rotation rate, these peaks are not a problem because, by the signal integration, the average impact of the ramp signal is mathematically null. However, for a FORS, the peaks can cause confusion and should be removed, but because these artefacts are deterministic (depending on the measured rotation rate), their elimination is problematic. Overall, there are two ways to solve this problem. The first, called “passive” by the authors, is related to the existing electronic part of the system adopted from FOG and uses a deramping procedure operating on Mini-SEED files with raw data. This procedure has been successfully used for a one-axis, as well as a three-axis FORS-blueSeis-3A (iXBlue, Cedex, France) [46]. This method uses the output signal from the system and correlates it with information about the signal driving the MIOC to remove the ramp peaks from the raw data (see Figure 3). The second one, named the “active” method, requires the reconfiguration of a field-programmable gate array (FPGA), where information about when the MIOC signal achieves a 2π value is known with one sampling accuracy. This sample could be removed and replaced by the interpolated value of the samples before and after it. In this way, the output data should be free of ramp peaks. A higher sampling rate of signal digitalisation is preferred for this method. Unfortunately, both methods discussed above were not possible to be implemented for the presented results of the FOS5 (see the existing ramp peaks at the filtered signal in Figure 2). However, they are being investigated for the currently constructed version of the FOS6.

### 3.2. Optimisation of Data Transfer Rate and File Size

The data transferred from a FORS should be in a Mini-SEED format, which is commonly used by the seismological society as an appropriate method of data transfer [47]. This format is also useful for implementation in free seismic analysis software, such as the SeisGram2K Seismogram Viewer (ALomax Scientific, Mouans-Sartoux, France) or others. Unfortunately, a critical parameter is the file size which should not be larger than 80–100 MB. Although Mini-SEED is well compressed, the general sampling of about 200 sps is the maximum rate for data registered over a 24-h period for a file size less than 100 MB. A sampling rate of 200 Hz is enough for a bandpass required by the RS, but it should be underlined that a much higher sampling rate is useful for several reasons, such as the ability to eliminate ramp peaks and data synchronization between FORS channels or between different FORSs. As the FOS5 natively uses a sampling of 1 ms (1000 Hz), one-hour data in Mini-SEED format is about 14 MB, which gives about 340 MB of data from a continuous recording of one day; this is too much for future investigation by a commercially available software. It should be noted that other solutions also use high sampling rates, for instance, iXblue uses a sampling rate of up to 380 kHz in blueSeis-3A [48]. It is, therefore, necessary to resample data down to 200 Hz, which, in turn, gives about 72 MB of Mini-SEED files from 24 h of recording for the FOS5.

**Figure 4:**
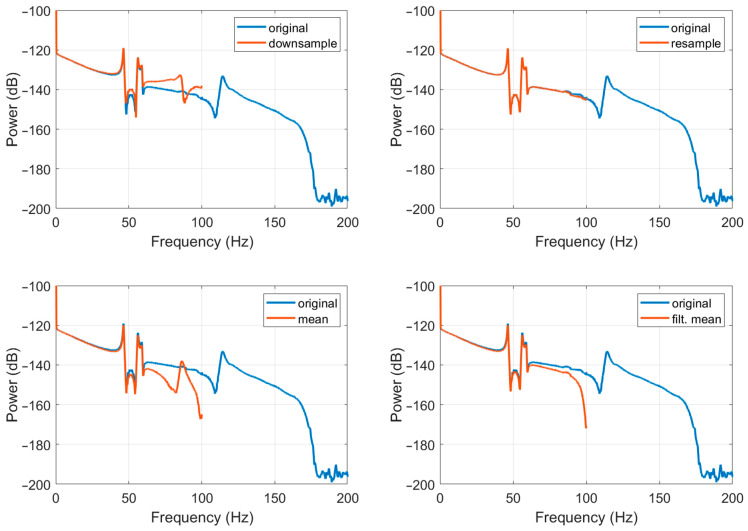
Comparison of the spectral accuracy of different investigated resampling methods based on a simulated spectral signal.

Of the many methods of data resampling [49], four methods have been investigated, including: simple “downsample”, “mean”, “filtered mean”, and “resample”. The “downsample” method produces a resampled signal by selecting the first sample and then every fifth sample. The “mean” method is quite straightforward as it is based on calculating the mean value of five consecutive samples. The “filtered mean” method introduces low-pass filtering (in this case, Kaiser window of the 100th order with parameter β set to five), followed by normalisation before applying the “mean” method. The “resample” method also introduces low-pass filtering (in this case, Kaiser window of the 100th order with parameter β set to five), followed by normalisation before applying the “downsample” method. The first two are easily computable and fast methods that are possible to be implemented directly in the data acquisition module, but they distort high-frequency signal components (above ~40 Hz). The “mean” method additionally smooths the signal and reduces the high-frequency peaks, as shown in the simulation in Figure 4. The latter two provide satisfactory results, as they give good reconstruction signal with small disturbances only in higher frequencies (above ~90 Hz for the “resample” method and above ~80 Hz for the “filtered mean” method, as shown in Figure 4). However, they use more complicated calculations and need longer (about one hour) datasets. Therefore, these methods can be implemented in the post-processing only.

**Figure 5:**
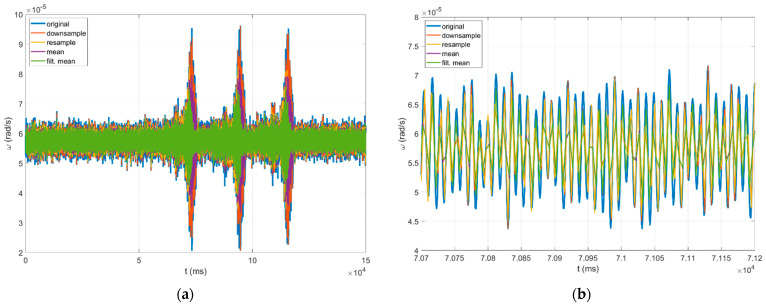
Seismogram of rotational events recorded by the FOS5-02 during the Fürstenfeldbruck experiment on 21 November 2019, for a series of three sweep sine vibrations generated by a special VibroSeis truck (peak force: 275 kN) provided by a TU Bergakadennie Freiberg: (**a**) recorded signal for a series of three sweep sine generations, and (**b**) a window magnifying the first of the events. Different colours show the results of the application of different resampling methods with their identification presented in the top-left window.

**Figure 6:**
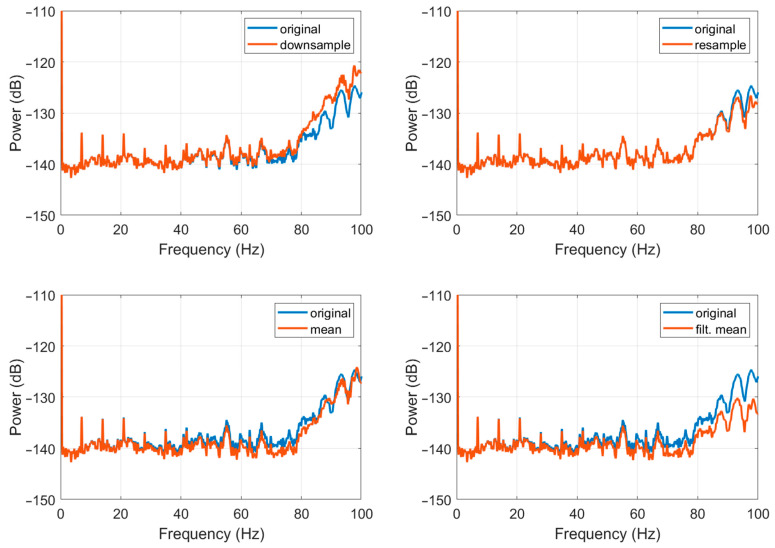
Comparison of the spectral accuracy of the different investigated resampling methods applied to a signal from Figure 5b.

The practical implementation of the above-described resampling methods from 1000 Hz to 200 Hz for the FOS5-02 is presented in Figure 5 and Figure 6, which is based on a seismogram recorded on 21 November 2019 during the huddle test in Fürstenfeldbruck, Germany, as described in detail in papers [30,31]. As one can see, the resample method gives the best results.

### 3.3. Automatic Elimination of Artefacts from Recorded Data

**Figure 7:**
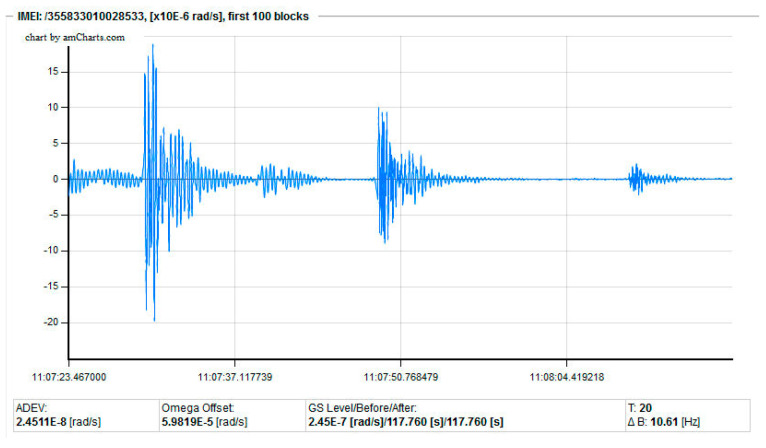
Example (screenshot from dedicated software) of a properly recorded rotational seismic event using the AFORS-1, which uses an open-loop configuration, installed at the seismological observatory in Książ Castel, Poland [34].

Seismic signals need to be recorded so they can be preserved and processed. A typical recorder consists of a digitizer connected to a computer, which can store all the data in a continuous form or store only the seismic events ([27], pp. 149–196). Especially for continuous monitoring systems, where the occurrence of expected phenomena should be recorded, the automatic start of recording data is crucial. Such situation exists in seismology when collecting seismic events, where a simple procedure to start data collection seems to be reasonable if the signal is higher than a certain level, as shown in Figure 7.

**Figure 8:**
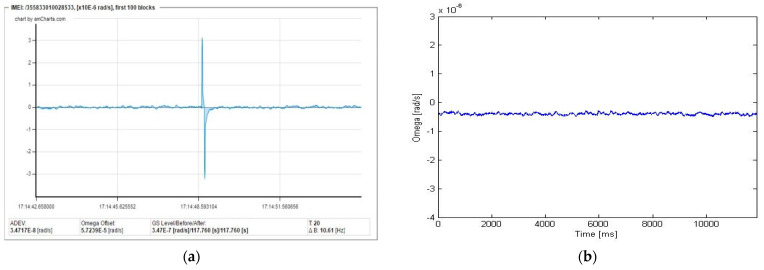
Seismogram (screenshot from dedicated software) recorded by the AFORS-1 at the Książ Castle seismic observatory, Poland: (**a**) registration of only artefacts, and (**b**) modification of the data collection procedure securing the elimination of artefacts [34].

However, if the ADCs used in the electronic part generate artefacts (single data with a random value over the noise level), the above recording procedure triggers an inefficient operation which can be classified as a false-positive case, as shown in Figure 8a. As can be seen, this is not a proper action, so a new and more complex algorithm for data collection must be used, such as the one shown in Figure 8b, which secures the correct elimination of the given artefacts and has been thoroughly described in our previous publication [34].

**Figure 9:**
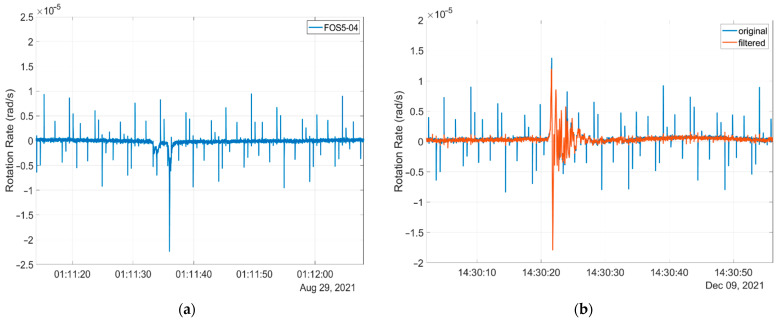
Seismogram recorded by the FOS5-04: (**a**) existence of a series of unrecognised artefacts, and (**b**) result of an application of the latest algorithm for artefact “dumping”.

Unfortunately, the correct choice of such algorithm is much more complicated because of a more complex structure of artefacts or other types of imperfections, which are easy to recognise visually, but harder to recognise automatically. As an example, Figure 9a presents a seismograph recorded recently in Książ, Poland, by our FOS5-04. As one can see, artefacts repeating within a period of about 0.6 s and with random amplitudes are observed, which source has not been yet correctly identified.

Proper elimination of the above disturbances is a big challenge. The obtained results are presented in Figure 9b as a filtering procedure with different power. The signal filtering applied is a two-step process. In the first phase, the detection of disturbances is carried out and unwanted “spikes” are distinguished from the actual measurement signal. At this stage of the work, it was decided to use an algorithm based on the following conditions. The samples are selected as a disturbance (“spike”) when (1) there is a local extremum of the signal; (2) the value exceeds the mean value of the modulus of the original signal (after removing the constant component) several times; and (3) in the immediate vicinity of the sample, there is no similar signal but of opposite sign. The algorithm has numerous parameters (e.g., maximum window length to calculate the mean value of the signal, and mean value of the signal module after removing the constant component) established experimentally. The algorithm parameters should also be adjusted to the sampling frequency. The methodology adopted in this way means that errors will be made both in omitting some disturbances and in eliminating correct areas. This is the basis for the further development of the algorithm or its replacement with a more advanced classification method. The second phase is the process of eliminating disturbances in the places indicated by the algorithm (along with the immediate surroundings). The elimination consists of replacing the pin with a constant signal with the average value of the signal from the window.

**Figure 10:**
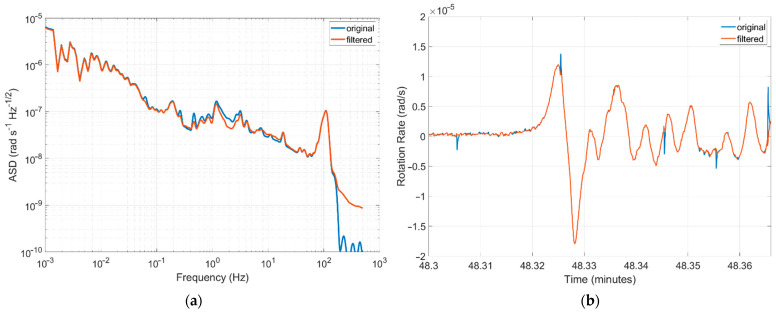
Accuracy of the applied method for the elimination of artefacts: (**a**) comparison of the ASD characteristics of the FOS5-04 with and without signal filtering, and (**b)** seismogram with rotational events and their disturbance by artefact filtering.

The authors’ investigation directed at using a proper algorithm to remove such artefacts can be regarded as still unsatisfactory. The best results show that it is possible to successfully dump most of them, as shown in Figure 9b and Figure 10a for the ASD characteristics. However, the existence of a seismic event means such an approach disturbs their characteristics (the same signal components are eliminated), as shown, for example, in Figure 10b.

## 4. Discussion

The authors’ experience in the research on fibre-optic rotational seismometers and seismographs for nearly 20 years has shown that signal processing should be numerically post-processed at every level of data investigation.

The first stage in the above procedure is the investigation of self-noise, as shown in Section 3.1. The obtained spectral characteristics of instruments as PSD or ASD are very noisy. For the above reason, all authors present the results after filtration, but information about the type of procedure and its parameters should be directly underlined, for instance, in our case, it is the commonly used Konno–Ohmachi filter with a smoothing coefficient of 40. At this stage of device investigation, there is a noticeable limitation due to the direct application in the FORS of the electronic solution prepared for the FOG, which introduces sharp spectral peaks at higher frequencies. Their elimination requires an implementation of the deramping procedure at post-processing level or modification of the FORS electronic part by reconfiguration of the FPGA structure.

The second stage is related to the suitable data preparation for the visualisation of the recorded rotational seismic events. In the present paper, the authors focused on two elements that are important from their point of view. The first is the suitable size of the data file transferred in Mini-SEED format. In the authors’ opinion, it should be data with 200 sps because such rate is sufficient for the 100 Hz upper frequency expected for the RS. However, since electronic parts use a much higher sampling rate (for the FOS5, it is 1000 Hz), a resampling method must be implemented. The investigated FOS5 devices use a resample method which is suitable, as shown in Section 3.2, but other solutions, such as the blueSeis-3A produced by iXblue, use a more complex solution [48]. The second element is a method for an automatic elimination of any artefacts observed in the data streams, which are caused by the limited accuracy of the DACs used or by unrecognised sources at the device installation. The solution to this problem is discussed in Section 3.3; however, the final solution still requires further research.

## Data Availability

Data available on request from corresponding author.
